# Emerging multidimensional biomarker system for cardiovascular-kidney-metabolic syndrome: from multi-omics integration to clinical artificial intelligence

**DOI:** 10.1186/s12933-026-03178-0

**Published:** 2026-04-26

**Authors:** Fei-Hong Li, Yuan-Yu Li, Yue Zhang, Liu Yang, Shao-Kang Pan, Dong-Wei Liu, Zhang-Suo Liu, Zhong-Xiuzi Gao, Peng Wu

**Affiliations:** 1 Department of Nephrology, The First Affiliated Hospital of Zhengzhou University, Zhengzhou, China; 2 State Key Laboratory of Metabolic Dysregulation & Prevention and Treatment of Esophageal Cancer, Tianjian Laboratory of Advanced Biomedical Sciences, Institute of Nephrology, Zhengzhou University, Zhengzhou, China; 3 Henan Province Research Center for Kidney Disease, Zhengzhou, China; 4 Key Laboratory of Precision Diagnosis and Treatment for Chronic Kidney Disease in Henan Province, Zhengzhou, China

**Keywords:** Cardiovascular-kidney-metabolic syndrome, Biomarkers, Multi-omics technologies, Artificial intelligence, Precision medicine

## Abstract

Cardiovascular-kidney-metabolic (CKM) syndrome is an emerging clinical entity that highlights the complex, bidirectional interplay among cardiovascular disease, chronic kidney disease, and metabolic disorders, representing a substantial and growing global health burden. This conceptualization marks a paradigm shift from viewing these conditions in isolation to understanding them as an interconnected disease continuum. Traditional biomarkers face significant limitations in the early detection, risk stratification, and precise management of CKM, necessitating a transition towards an integrated framework that captures its multisystem nature. This review systematically outlines an emerging multidimensional biomarker system encompassing key pathological axes such as metabolism, immuno-inflammation, oxidative stress, and biological aging, offering refined risk assessment beyond conventional metrics. The development of this system is propelled by revolutionary platforms, including accessible sampling techniques (e.g., dried blood spots), advanced in vitro models (e.g., multi-organ-on-a-chip), and multi-omics technologies. These platforms not only facilitate a deeper dissection of the heterogeneous origins and inter-organ crosstalk in CKM but also accelerate the discovery and validation of novel biomarkers. Concurrently, artificial intelligence serves as a pivotal tool for clinical translation, effectively integrating high-dimensional data to transform complex molecular profiles into actionable clinical insights. By enabling the construction of dynamic risk prediction and decision-support systems, this review charts a pathway toward proactive, individualized, and precise prevention and management of CKM syndrome.

## Introduction: clinical challenges of CKM syndrome and the paradigm shift in biomarkers

Cardiovascular-kidney-metabolic (CKM) syndrome is an integrative clinical concept formally introduced by the American Heart Association (AHA) in 2023 [1, 2]. It describes the complex, bidirectional, and mutually reinforcing pathophysiological interactions among cardiovascular disease (CVD), chronic kidney disease (CKD), and metabolic disorders, including obesity, type 2 diabetes, and insulin resistance (IR) [3, 4]. This conceptual advancement signifies a critical evolution in medical understanding, moving from a historically siloed view of cardiac, kidney, and metabolic diseases towards recognizing them as a dynamically evolving, multi-organ interactive continuum. The global burden of CKM syndrome is profound. Large-scale epidemiological studies reveal a high prevalence of various CKM stages among adults in the United States [5–7], with advanced stages (3–4) strongly associated with a steep increase in all-cause and cardiovascular mortality risks [8–14]. In rapidly developing nations like China, the prevalence of CKM is also rising significantly, driven by population aging and lifestyle changes [15]. Furthermore, Global Burden of Disease studies indicate that conditions central to CKM (e.g., ischemic heart disease, stroke, diabetes, CKD) impose the heaviest burden in middle sociodemographic index regions, a trend projected to persist through 2046 [16]. This state of multisystem comorbidity severely impairs quality of life [17] and poses a major socioeconomic challenge [16], underscoring the urgent need for early identification, accurate risk stratification, and proactive intervention [16, 18].

In clinical practice, CKM assessment has long relied on traditional biomarkers such as fasting blood glucose, glycated hemoglobin (HbA1c), lipid profiles (LDL-C, HDL-C, TG), and serum creatinine (for estimating glomerular filtration rate, eGFR). However, these indicators have notable limitations. First, as downstream markers of established pathology, they often exhibit a lag, typically becoming abnormal only after substantial organ damage or overt clinical manifestations, thus failing to capture early, potentially reversible pathophysiological disturbances [19]. Second, their mechanistic specificity and discriminatory power are often insufficient. CKM exhibits significant heterogeneity, with different patients presenting dominant phenotypes such as predominant cardiac dysfunction, kidney decline, or metabolic dysregulation [20, 21]. Traditional biomarkers inadequately reflect the core driving mechanisms behind this heterogeneity, including the severity of insulin resistance [22], the degree of systemic inflammation [14, 23–25], the state of oxidative stress [26], or the pace of biological aging [27–29]. Consequently, they may fail to effectively distinguish subgroups with divergent risks within the same diagnostic stage [30]. Therefore, constructing a novel biomarker system that comprehensively reflects the core pathological mechanisms of CKM is imperative to overcome current clinical management bottlenecks. This field has witnessed rapid progress in recent years. On one hand, novel composite indices derived from routine clinical data—such as the estimated glucose disposal rate [27, 31–33] and the triglyceride-glucose index and its derivatives (TyG-BMI, TyG-WHtR, etc.) [22, 34–37]—have demonstrated considerable clinical promise due to their accessibility, low cost, and enhanced predictive value, and are beginning to be incorporated into updated risk assessment frameworks [38–40]. On the other hand, the deep integration of multi-omics technologies (metabolomics, proteomics, epigenomics) [19, 41–43], portable diagnostic platforms (e.g., dried blood spot technology [44]), and artificial intelligence (AI) algorithms is driving a transformative shift in biomarker discovery and application [28, 45–50]. This evolution represents not merely a technological advancement but a fundamental update in disease management philosophy [51], moving from single, static measurements towards multi-dimensional, dynamic, and network-based biomarker systems analysis.

This review aims to systematically synthesize the latest evidence on emerging biomarkers in CKM, attempting to integrate these disparate advances into a coherent, multi-dimensional biomarker framework. We will elucidate the value of this framework in early screening, precise staging, prognostic risk assessment, and individualized intervention for CKM. Furthermore, we will explore the future landscape of CKM clinical management—propelled by AI and advanced diagnostic technologies—toward a paradigm of precision, proactivity, and equitable care.

## The global landscape of CKM syndrome: epidemiology and disease burden

CKM syndrome has evolved from a specialized clinical concept into a pervasive public health crisis with global ramifications. Analyses of recent US National Health and Nutrition Examination Survey (NHANES) data paint a concerning picture. After age standardization to the 2000 US standard population to account for differences in age distribution across survey cycles, the estimated prevalence of each CKM stage among US adults is as follows: stage 0 (no CKM risk factors) accounts for 13.6% of the population; stage 1 (excess or dysfunctional adiposity) accounts for 29.9%; stage 2 (concurrent metabolic risk factors or chronic kidney disease) accounts for 43.7%; stage 3 (subclinical cardiovascular disease) accounts for 4.7%; and stage 4 (established clinical cardiovascular disease) accounts for 8.1%. Collectively, advanced stages (3 and 4) constitute 12.8% of the adult population, indicating that tens of millions of Americans face a substantially elevated risk of cardiovascular events and death [5–7, 18]. This burden is both universal and growing worldwide. Large-scale cohort studies from Asia corroborate this severity. A decade-long prospective cohort study in China demonstrated a significant gradient between CKM stages and incident cardiovascular events, with stage 4 patients facing a nearly six-fold higher risk compared to stage 0 [52]. In South Korea, a nationwide study (2011–2021) showed a significant annual increase of 3.2% in the prevalence of stage 4 CKM, alongside a decline in stage 0 prevalence [53]. Beyond Asia, data from the UK Biobank—one of the largest European prospective cohorts—confirm a graded association between CKM stages and all-cause and cause-specific mortality, with stage 4 conferring a more than twofold increased risk of all-cause mortality compared with stage 0 [54]. These regional trends align with macro-level analyses forecasting a persistently increasing global disease burden for key CKM components, including diabetes, obesity, CKD, and CVD [16, 55].

The detrimental impact of CKM extends beyond high prevalence to its lethal prognosis. Multiple large-scale studies consistently confirm CKM stage as a powerful predictor of all-cause and cardiovascular mortality. A prospective US cohort of over 34,000 adults showed escalating all-cause mortality risks with ascending CKM stages. Compared to stage 0, the hazard ratios for stages 2, 3, and 4 were 1.43, 2.75, and 3.02, respectively, with cardiovascular mortality risk in stage 4 soaring to 10.5-fold higher [9]. Quantified in terms of life expectancy, a 50-year-old individual in stage 4 has a life expectancy shortened by approximately 12.4 years compared to someone in stages 0–1 [13]. From a population-attributable risk perspective, advanced CKM stages (3–4) account for a population-attributable fraction of 45.3% for cardiovascular deaths [10]. Similarly, a Chinese prospective study of nearly 100,000 individuals revealed a strong dose–response relationship between CKM stage and all-cause mortality, particularly pronounced in those under 60 [11]. Furthermore, CKM syndrome is strongly associated with end-stage kidney disease (ESKD) risk; a Taiwanese study of over 510,000 individuals found stage 4 patients had a more than tenfold higher risk of ESKD [8], underscoring its severe kidney impact. This refinement in risk assessment is driving the application of personalized treatment and prompting a re-evaluation of kidney disease's central role within the CKM spectrum [56, 57]. Beyond direct mortality, CKM syndrome impairs quality of life through multi-system complications. Research indicates a significant association between CKM and cognitive decline/dementia risk [48, 58–60]. A bidirectional relationship exists with mental health; poor CKM health is linked to higher risks of depression and anxiety, amplified by social isolation [61–63]. Conversely, depression is an independent predictor of CKM progression and elevated mortality [64–66], prompting efforts to develop models predicting depression risk in advanced CKM [67]. Additionally, poor CKM health is associated with an increased risk of psoriasis and reduced life expectancy in affected patients [68, 69].

The distribution and progression of CKM are profoundly influenced by social determinants of health, sex, race and ethnicity, and environmental exposures. Adverse socioeconomic conditions (e.g., low income, food insecurity) are independently associated with advanced CKM [18, 70, 71], necessitating their integration into management [72]. While men have a higher prevalence of stage 3 CKM, women exhibit greater mortality risks at the same stage [73], suggesting potential pathophysiological differences potentially related to sex-specific lifespan factors [74]. Racial and ethnic differences further shape CKM epidemiology. In the United States, non-Hispanic Black adults have the highest prevalence of advanced CKM stages (stages 3–4), followed by non-Hispanic White and Hispanic adults, with disparities persisting after adjustment for age, sex, and socioeconomic factors [7, 18]. These differences reflect not only genetic ancestry but also the cumulative effects of structural inequities—including differential access to preventive care, neighborhood-level socioeconomic deprivation, and lifelong exposure to psychosocial stressors—that disproportionately burden historically marginalized populations [72]. Moreover, among individuals with advanced CKM, those from racial and ethnic minority groups experience higher mortality rates compared with non-Hispanic White individuals, even after accounting for income and education [71]. Environmental exposures also play a critical role [75, 76]; ambient air pollution and household solid fuel use independently elevate advanced CKM risk [77], while exposures to metals like cadmium and plasticizers can drive CKM through metabolic, inflammatory, and fibrotic pathways [78–81].

In summary, epidemiological data clearly demonstrate that CKM syndrome is a prevalent and escalating global health threat. Its stages accurately correspond to a gradient of increasing mortality and complication risks, with its burden disproportionately borne by socioeconomically disadvantaged groups, demanding both comprehensive and targeted prevention and management strategies.

## Bibliometric analysis of cardiovascular-kidney-metabolic syndrome research

To provide an objective, high-level overview of the research landscape and evolving trends in CKM syndrome—complementing the narrative synthesis that follows—we conducted a bibliometric analysis using the Web of Science Core Collection. This database was selected as the data source for this analysis due to its standardized data export format (fully compatible with widely used bibliometric software) and its long citation time frame (dating back to 1900)—both of which support reproducible network analyses [82]. The search was performed up to November 18, 2025, restricted to English-language articles and reviews. The search strategy employed topic-specific terms related to CKM syndrome, identifying 310 relevant publications. Data analysis and visualization were performed using Microsoft Office Excel, SCImago Graphica, VOSviewer, CiteSpace, and the R package "bibliometrix".

**Fig. 1:**
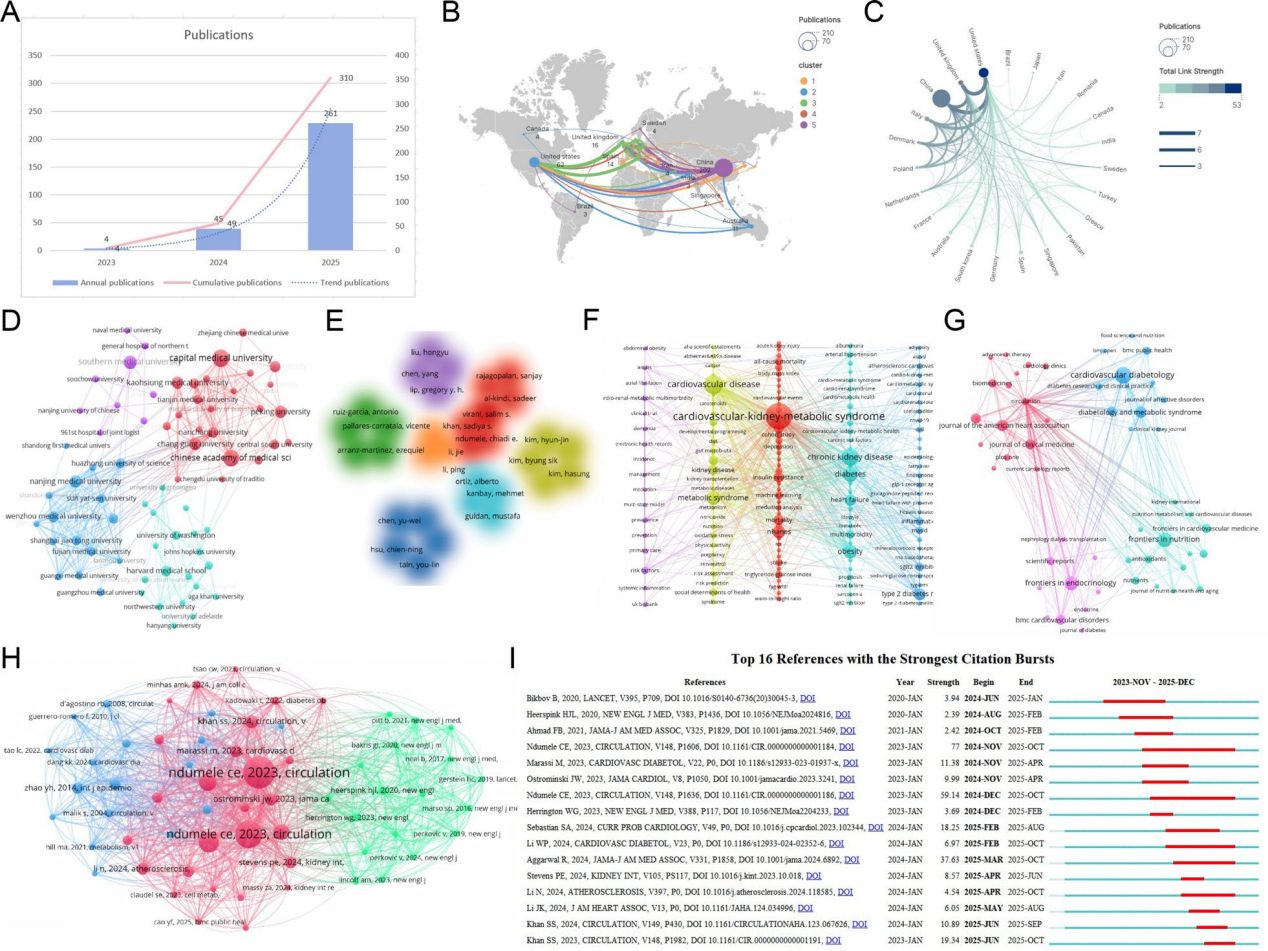
Bibliometric analysis of research on Cardiovascular-Kidney-Metabolic syndrome. **A** Annual and cumulative publication trends, illustrating a marked increase in research output. **B** Global distribution of contributions, with circle size proportional to publication volume and color intensity indicating collaboration density. **C** Country collaboration network, highlighting the leading roles of China, the United States, and European nations. **D** Institutional collaboration network, showing prominent contributions from academic and medical research centers. **E** Author collaboration heatmap, identifying key researchers and their collaborative relationships. **F** Keyword co-occurrence map, revealing core research themes such as cardiovascular disease, chronic kidney disease, and metabolic syndrome. **G** Citation relationships among journal sources, emphasizing the influential role of specialized cardiometabolic journals. **H** Co-citation network of references, anchored by seminal American Heart Association statements and guidelines. **I** Citation burst analysis, identifying highly influential references and emerging research fronts.

A marked and accelerating growth in annual publications was observed, signaling rapidly expanding global research interest (Fig. 1A). Geographically, China and the United States were the dominant contributors (Fig. 1B). China led in productivity (202 documents), whereas the United States, with fewer publications (62 documents), demonstrated greater total link strength, reflecting a more extensive international collaborative network (Fig. 1C). The United Kingdom, Italy, and Spain also ranked among the top five productive countries. At the institutional level, Capital Medical University and the Chinese Academy of Medical Sciences & Peking Union Medical College were the most productive (Fig. 1D). Chinese institutions featured prominently, indicating concentrated research efforts within the country. Notably, Harvard Medical School, though ranking tenth in productivity, received high citation counts, underscoring the significant impact of its output. Among individual authors, Hsu, Chien-Ning and Tain, You-Lin led in productivity, while Khan, Sadiya S. and Tuttle, Katherine R. received the highest citation counts, denoting their considerable influence (Fig. 1E). Keyword co-occurrence analysis revealed "Cardiovascular-Kidney-Metabolic Syndrome" as the most frequent term, followed by "Cardiovascular Disease," "Chronic Kidney Disease," and "Metabolic Syndrome," underscoring the field's interdisciplinary focus (Fig. 1F). *Cardiovascular Diabetology* published the highest number of articles (23 documents) and ranked highly in total link strength, highlighting the pivotal role of specialized cardiometabolic journals (Fig. 1G). The journal *Circulation* was the most influential source, with only 4 documents amassing 1571 citations (Fig. 1H). The most co-cited references were dominated by seminal American Heart Association statements and guidelines, particularly the works by Ndumele et al. (2023) and Khan et al. (2023–2024), which establish the foundational framework for CKM syndrome (Fig. 1I).

In conclusion, this bibliometric analysis portrays a rapidly evolving, collaborative research domain with a clear conceptual focus on cardiorenal-metabolic interrelationships, anchored in high-impact clinical guidelines. The trends identified here—particularly the central role of seminal AHA statements, and the key themes reflected in keyword co-occurrence—provide a foundation for the detailed examination of emerging biomarkers, technological platforms, and clinical applications that follow in the subsequent sections.

## Beyond the conventional: the evolving landscape of emerging biomarkers

**Fig. 2:**
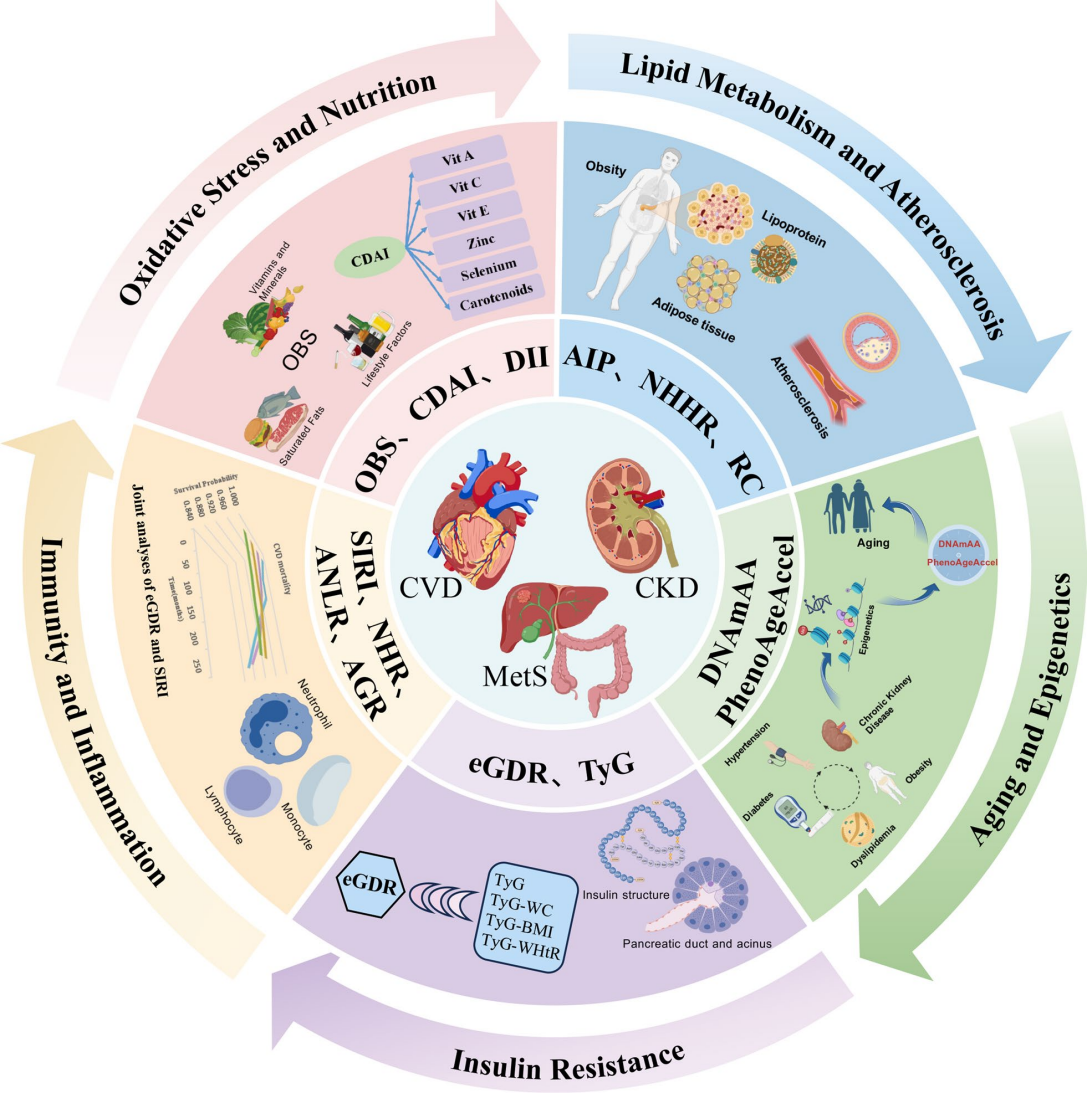
A multidimensional biomarker framework for Cardiovascular-Kidney-Metabolic syndrome. The diagram illustrates five interconnected pathological axes central to CKM syndrome, each associated with key emerging biomarkers: Insulin resistance (eGDR, TyG and its derivatives TyG-BMI, TyG-WC, TyG-WHtR); Immunity and inflammation (SIRI, NHR, ANLR and AGR); Oxidative stress and nutrition (OBS, CDAI and DII); Lipid metabolism and atherosclerosis (AIP, NHHR and RC); Aging and epigenetics (DNAmAA and PhenoAgeAccel). These dimensions collectively enable a more comprehensive and earlier assessment of CKM risk and progression beyond conventional biomarkers. The figure was created with BioRender.com. eGDR, estimated glucose disposal rate; TyG, triglyceride-glucose index; TyG-BMI, triglyceride-glucose index combined with body mass index; TyG-WC, triglyceride-glucose index combined with waist circumference; TyG-WHtR, triglyceride-glucose index combined with waist-to-height ratio; SIRI, systemic immune-inflammation index; NHR, neutrophil to high-density lipoprotein cholesterol ratio; ANLR, albumin-to-neutrophil/lymphocyte ratio; AGR, albumin-to-globulin ratio; OBS, oxidative balance score; CDAI, composite dietary antioxidant index; DII, dietary inflammatory index; AIP, atherogenic index of plasma; NHHR, non-high-density lipoprotein cholesterol to high-density lipoprotein cholesterol ratio; RC, remnant cholesterol; DNAmAA, DNA methylation age acceleration; PhenoAgeAccel, phenotypic age acceleration

The pathological core of CKM syndrome lies in the multisystem interplay of several key mechanisms. Insulin resistance, chronic inflammation, and oxidative stress form a "malignant triad" that drives disease initiation and progression. Insulin resistance not only underpins metabolic dysregulation but also directly promotes vascular endothelial dysfunction, increased kidney sodium reabsorption, and sympathetic nervous system activation [2]. The resulting metabolic disorder is a primary instigator of cardiovascular and kidney fibrosis [83]. This process interacts with and is exacerbated by chronic, low-grade inflammation stemming from innate immune system activation. Pro-inflammatory cytokines (e.g., IL-6, TNF-α) further aggravate insulin resistance and directly damage cardiovascular and kidney tissues [23]. Concurrently, excessive reactive oxygen species (ROS) generation from oxidative stress impairs nitric oxide bioavailability—leading to vascular dysfunction—and activates inflammatory pathways, inducing cellular apoptosis and fibrosis, thereby forming a vicious cycle that amplifies tissue injury [84, 85]. Additionally, disorders of lipid metabolism, characterized by an elevated atherogenic index of plasma and increased remnant cholesterol, independently propel CKM toward advanced stages through pro-inflammatory and pro-atherosclerotic mechanisms [49, 86, 87]. The long-term consequence of these core mechanisms is accelerated biological aging. Studies indicate that epigenetic age acceleration is significantly associated with advanced CKM stages and mortality [88], while insulin resistance itself is closely linked to accelerated aging [27]. This suggests that assessing an individual's "biological age" may more precisely quantify the cumulative health damage inflicted by CKM. Given the systemic, interactive, and cumulative nature of this pathological network, traditional single biomarkers are inadequate for precise assessment. There is an urgent need for a novel biomarker system capable of multi-dimensional quantification [24]. This section systematically reviews emerging biomarkers across five key dimensions—insulin resistance, inflammation, oxidative stress, lipid metabolism, and aging—which together form a new framework for earlier and more comprehensive assessment of CKM risk and prognosis (Fig. 2 and Table 1).

**Table 1:** Emerging multidimensional biomarkers in cardiovascular-kidney-metabolic (CKM) syndrome

Category	Name	Physiological significance	Impact on CKM disease	References
Insulin Resistance	Estimated Glucose Disposal Rate (eGDR)	Reflects whole-body insulin sensitivity; lower values indicate worse insulin resistance	In CKM stages 0–3, lower eGDR is independently associated with higher risks of CVD incidence, all-cause mortality, and CVD mortality. It improves predictive performance beyond traditional risk scores	[27, 31–33, 89]
	Triglyceride-Glucose Index (TyG)	Represents insulin resistance and lipid-glucose metabolism dysregulation	Higher TyG is independently associated with increased risks of CVD incidence and mortality in CKM stages 0–3	[22, 34–36]
	TyG-Body Mass Index (TyG-BMI)	Combines insulin resistance with overall adiposity	Strongly predicts CVD incidence and mortality in individuals with CKM stages 0–3	[34, 36, 90]
	TyG-Waist Circumference (TyG-WC)	Reflects central obesity and insulin resistance	Demonstrates superior predictive performance for CVD and mortality in CKM populations compared to TyG alone	[22, 34–36]
	TyG-Waist-to-Height Ratio (TyG-WHtR)	Adjusts central obesity for height, better reflects visceral fat	Strongly associated with stroke and CVD mortality in CKM stages 0–3	[22, 34, 36]
	TyG-A Body Shape Index (TyG-ABSI)	Captures both metabolic and body shape risk	Predicts all-cause and CVD mortality in CKM stages 0–3	[91, 92]
	TyG-Chinese Visceral Adiposity Index (TyG-CVAI)	Tailored for Asian populations, reflects visceral adiposity and metabolic risk	Strongly predicts stroke and CVD risk in Asian CKM populations	[92]
	Cumulative TyG (cumTyG)	Captures long-term metabolic dysregulation	Predicts frailty and CVD risk in longitudinal CKM studies	[93, 94]
Immunity and Inflammation	Systemic Immune-Inflammation Index (SIRI)	Reflects systemic inflammatory and immune status	Elevated SIRI is associated with higher CVD incidence, all-cause mortality, CVD mortality, and worsening renal function (WRF); mediates the depression–mortality link	[14, 25, 32, 46, 95]
	Neutrophil to HDL-C Ratio (NHR)	Integrates inflammation and lipid metabolism	Higher NHR is linked to increased prevalence of CKM syndrome, CVD mortality, and all-cause mortality	[96]
	Albumin-to-Neutrophil/Lymphocyte Ratio (ANLR)	Combines nutritional status (albumin) with inflammatory burden	Lower ANLR predicts higher all-cause and CVD mortality in advanced CKM (stages 3–4)	[97]
	Albumin-to-Globulin Ratio (AGR)	Reflects nutritional and inflammatory status	Lower AGR is associated with higher all-cause mortality, CVD mortality, and CKM severity	[98]
Oxidative Stress and Nutrition	Oxidative Balance Score (OBS)	Reflects overall oxidative stress balance from diet and lifestyle	Higher OBS is associated with lower odds of advanced CKM stages and reduced all-cause/CVD mortality. It also mediates the effects of inflammation and frailty on mortality	[26, 99–102]
	Composite Dietary Antioxidant Index (CDAI)	Indicates dietary antioxidant capacity	Higher CDAI is linked to lower odds of advanced CKM stages	[102–104]
	Dietary Inflammatory Index (DII)	Reflects pro-inflammatory dietary patterns	Higher DII is associated with higher CKM prevalence, CKD risk, advanced CKM stages, and mortality	[28, 62, 104–106]
Lipid Metabolism & Atherosclerosis	Atherogenic Index of Plasma (AIP)	Reflects atherogenic lipid profile	Higher AIP predicts increased risk of CVD, stroke, and mortality in CKM stages 0–3	[49, 107–112]
	Non-HDL-C/HDL-C Ratio (NHHR)	Represents atherogenic lipid burden	Associated with higher prevalence of CKM syndrome	[113]
	Remnant Cholesterol (RC)	Represents triglyceride-rich lipoprotein cholesterol	Elevated RC is linked to advanced CKM stages, incident CVD, and frailty progression	[86, 87]
	Residual Cholesterol Inflammation Index (RCII)	Integrates RC with inflammatory markers; captures lipid-inflammation interplay	Strongly associated with frailty progression in early CKM	[94]
Aging and Epigenetics	DNA Methylation Age Acceleration (DNAmAA)	Reflects biological aging influenced by genetics and environment	Accelerated DNAmAA (especially GrimAA) is associated with advanced CKM stages and higher mortality; mediates diet–CKM and environmental exposure–CKM links	[29, 88]
	Phenotypic Age Acceleration (PhenoAgeAccel)	Indicates biological aging from clinical profiles	Higher PhenoAgeAccel mediates the relationship between eGDR and mortality; mediates periodontitis–CKM association and predicts mortality	[10, 27, 28, 114]
	Aging Clocks (e.g., Klemera-Doubal, PhenoAge)	Integrative measure of biological aging and organ dysfunction	Enhances CKM staging and predicts dementia and mortality	[28, 29]

### Insulin resistance

Despite its established role as a central pathophysiological driver of CKM, insulin resistance is not captured by conventional laboratory biomarkers. The gold standard for its assessment, the hyperinsulinemic-euglycemic clamp, is invasive, and confined to research settings. Surrogate measures such as fasting insulin or the homeostatic model assessment of insulin resistance (HOMA-IR) require insulin assays that are not universally available in routine clinical laboratories, particularly in resource-limited settings. Consequently, insulin resistance remains under-recognized in clinical practice, representing a critical gap in CKM risk assessment. This gap has motivated the development of accessible surrogate indices derived from routine clinical data—specifically, the estimated glucose disposal rate (eGDR) and the triglyceride-glucose (TyG) index and its derivatives—which enable practical evaluation of insulin resistance without requiring specialized testing.

#### Estimated glucose disposal rate (eGDR)

eGDR is a robust, non-insulin-requiring indicator of peripheral insulin sensitivity, calculated from waist circumference (WC), hypertension status, and HbA1c. The formular is: eGDR (mg/kg/min) = 21.25 − [0.09 × WC (cm)] − [3.29 × hypertension (yes = 1, no = 0)] − [0.09 × HbA1c (%)], with lower values indicating more severe insulin resistance [33, 89]. In CKM populations, eGDR has demonstrated superior predictive value for adverse outcomes. Large-scale prospective cohort studies confirm a significant inverse correlation between eGDR and both all-cause and CVD mortality in individuals with CKM [27, 31]. Furthermore, integrating eGDR with inflammation markers (e.g., the Systemic Inflammation Response Index, SIRI) can further refine mortality risk stratification within this population [32]. Critically, the predictive performance of eGDR surpasses that of other surrogate markers of insulin resistance. In the China Health and Retirement Longitudinal Study (CHARLS) cohort, eGDR outperformed the triglyceride-glucose (TyG) index and its obesity-combined derivatives (e.g., TyG-waist circumference [TyG-WC], TyG-body mass index [TyG-BMI]) in predicting incident cardiovascular events, including heart disease and stroke [33]. Similarly, analysis of the UK Biobank cohort showed that eGDR independently predicted future cardiovascular events in individuals with CKM and provided incremental predictive value to the established PREVENT risk equations [89]. Comparative evaluations using machine learning models have corroborated that among various IR surrogates, eGDR holds the highest predictive value for cardiovascular events in CKM, and its incorporation significantly enhances model performance [33]. These findings establish eGDR as a robust and practical biomarker for risk management in CKM.

#### Triglyceride-glucose index and its derivatives (TyG-BMI, TyG-WC, TyG-WHtR)

TyG index is another widely validated surrogate marker for IR. However, the TyG index alone does not account for obesity, particularly central adiposity—a core driver of CKM. Therefore, derivative indices that combine the TyG index with measures of obesity provide a more comprehensive assessment of metabolic abnormality risk and typically exhibit superior predictive performance compared to the TyG index alone [22, 36]. Multiple large cohort studies have demonstrated the excellent performance of these composite indices in predicting long-term mortality among individuals with CKM [34–36]. In the NHANES cohort, TyG-BMI, TyG-WC, and TyG-waist-to-height ratio (TyG-WHtR) were all significantly and positively associated with risks of all-cause and cardiovascular mortality in individuals at CKM stages 0–3, with TyG-WC and TyG-WHtR showing the strongest associations [35]. The combination of TyG with the A Body Shape Index (TyG-ABSI) has also proven to be independently associated with all-cause and cardiovascular mortality risks in the CKM population [91]. Further research indicates that dynamic monitoring of TyG changes or its combination with other obesity phenotype indicators can effectively predict stroke risk in CKM. A CHARLS-based study found that cumulative TyG (cumTyG) was linearly and positively associated with stroke risk, suggesting that long-term monitoring aids in early identification of high-risk individuals [93]. Similarly, a TyG derivative index combined with the Chinese visceral adiposity index (TyG-CVAI) also showed superior predictive value for stroke risk in this population [92]. In summary, when assessing metabolic risk in CKM, it is crucial to simultaneously consider both insulin resistance and obesity phenotypes, especially central adiposity.

### Immunity and inflammation

Chronic low-grade inflammation is a central pathological mechanism underlying the development of CKM syndrome, connecting metabolic disorders, organ damage, and fibrotic processes [24]. While traditional inflammatory markers like C-reactive protein hold value, emerging composite indices that integrate various immune cells or biochemical parameters demonstrate more refined discriminatory power in revealing inflammatory burden and prognosis in CKM.

#### Systemic immune-inflammation index (SIRI) and neutrophil to high-density lipoprotein cholesterol ratio (NHR)

SIRI, calculated from neutrophil, monocyte, and lymphocyte counts, provides a comprehensive assessment of systemic immune-inflammatory status. Elevated SIRI is independently associated with an increased risk of CVD incidence in individuals with CKM [95] and is a powerful predictor of mortality. NHANES-based studies show that higher SIRI levels are significantly associated with all-cause and CVD mortality in individuals at CKM stages 0–3 [32]. Its predictive capability is highlighted in joint analyses with insulin resistance markers like eGDR; individuals with both low eGDR and high SIRI exhibit the highest mortality risk, revealing a synergistic effect [32]. Machine learning models have also identified SIRI as a key feature for predicting CKM risk [46]. NHR ingeniously links innate immune response with lipid metabolism. A high NHR is not only independently associated with the prevalence of CKM syndrome but also effectively predicts cardiovascular mortality in this population. Studies show that for every one standard deviation increase in NHR, cardiovascular mortality risk in CKM increases by 31% [96]. This association likely stems from the combination of uncontrolled inflammation (represented by neutrophils) and dysfunctional high-density lipoprotein (HDL), which jointly exacerbate atherosclerosis and organ damage.

#### Albumin-inflammation integrated indices

The malnutrition-inflammation complex is common in advanced CKM and is closely associated with poor prognosis. Hypoalbuminemia itself is an independent risk factor for death in CKM [115]. The Albumin-to-Neutrophil/Lymphocyte Ratio (ANLR) and the Albumin-to-Globulin Ratio (AGR) combine nutritional status (albumin) with inflammation (NLR or globulin), with ANLR calculated as Albumin/(neutrophil count/lymphocyte count) and AGR calculated as Albumin/(total protein − Albumin), providing a more comprehensive assessment of homeostatic imbalance. Research confirms that in advanced CKM (stages 3–4), a lower ANLR is an independent risk factor for all-cause and cardiovascular mortality. After propensity score matching, patients with ANLR below 1.04 had approximately twofold and 1.5-fold higher risks of cardiovascular and all-cause mortality, respectively [97]. Restricted cubic spline analysis revealed an L-shaped relationship between ANLR and mortality, emphasizing the importance of maintaining low inflammation alongside good nutritional status. Similarly, AGR shows a significant inverse correlation with mortality in CKM. This association varies by outcome: AGR exhibits a nonlinear negative correlation with all-cause mortality (inflection point at 1.26) but a linear negative correlation with cardiovascular mortality. Patients in the highest AGR quartile had 45% and 53% lower risks of all-cause and cardiovascular mortality, respectively [98]. These integrated indices capture key pathophysiological processes driven by inflammatory consumption and declining nutritional reserves in chronic disease states, providing stronger prognostic information than single markers.

### Oxidative stress and nutrition

Oxidative stress serves as a critical bridge connecting metabolic dysregulation, inflammatory responses, and end-organ damage in CKM. Excessive ROS directly damage cellular components and exacerbate disease progression. Traditional single oxidative markers suffer from limitations such as poor stability and insufficient reflection of overall systemic status. Emerging composite scoring systems and dietary pattern assessments offer a more holistic perspective for evaluating the body's oxidative-reductive balance.

#### Oxidative balance score (OBS) and composite dietary antioxidant index (CDAI)

OBS is a systematic tool that quantifies overall oxidative stress load by integrating dietary antioxidants, pro-oxidants, and lifestyle factors (Table 2). A higher score indicates a greater antioxidant advantage. Large-sample analyses based on NHANES data consistently confirm that a higher OBS is significantly associated with lower CKM risk and severity. Studies show an inverse correlation between OBS and CKM stage [26], with individuals in the highest OBS quartile having a 23% lower risk of advanced CKM (stages 3–4) [99]. Another study confirmed that lower OBS was independently associated with CKM stage progression and increased mortality risks [100]. Detailed analyses revealed varying contributions from different OBS components, with magnesium, vitamin B6, and physical activity as protective factors, while smoking (cotinine) was a significant risk factor [100]. In longitudinal follow-up, a higher OBS predicted better survival in both non-advanced and advanced CKM patients [101]. Mediation analysis suggested that inflammatory markers partially mediate the association between OBS and advanced CKM risk, explaining a pathway through which improving oxidative balance alleviates inflammatory burden [101]. CDAI focuses on assessing the combined intake of six key antioxidant nutrients. In NHANES data, a higher CDAI score similarly demonstrated a strong protective effect against advanced CKM, with the highest quartile having 30% lower odds [103]. Restricted cubic spline analysis revealed a nonlinear inverse relationship [103]. In a large prospective cohort, an antioxidant diet (CDAI ≥ 0) was confirmed to reduce CKD risk, particularly in early CKM stages [104].

**Table 2:** Components of oxidative balance score and aging biomarkers in cardiovascular-kidney-metabolic syndrome

Biomarker/Score	Components/Elements	References
Oxidative Balance Score (OBS)	Antioxidants (15 items): dietary fiber, β-carotene, riboflavin (vitamin B2), niacin, vitamin B6, total folate, vitamin B12, vitamin C, vitamin E, calcium, magnesium, zinc, copper, selenium, physical activity	[97–99, 116]
	Pro-oxidants (5 items): total fat, iron, body mass index (BMI), alcohol consumption, serum cotinine (smoking)	
DNA Methylation Age Acceleration (DNAmAA)	Integrates DNA methylation levels at specific CpG sites with plasma proteins (cystatin C, growth differentiation factor-15, adrenomedullin, β-2-microglobulin, leptin, plasminogen activator inhibitor-1, tissue inhibitor metalloproteinases-1, C-reactive protein, hemoglobin A1c) and smoking pack-years	[86]
Phenotypic Age Acceleration (PhenoAgeAccel)	Calculated as the residual from regressing phenotypic age on chronological age, where phenotypic age is derived from nine clinical biomarkers: albumin, creatinine, glucose, C-reactive protein, alkaline phosphatase, lymphocyte percentage, mean cell volume, red blood cell distribution width, white blood cell count, combined with chronological age	[10, 27, 28, 117]

#### Dietary inflammatory index (DII)

DII measures the overall inflammatory potential of the diet. In the NHANES population, a higher DII score (more pro-inflammatory diet) was independently associated with a higher prevalence of CKM [105, 106]. This association was consistent across demographic subgroups, with alcohol intake identified as a strong driver [105]. Among individuals with established CKM, a higher DII score was also significantly associated with an increased risk of depressive symptoms, showing a J-shaped curve relationship [62]. Mediation analysis indicated that metabolic syndrome mediated 4.44% of this association [62]. Notably, combining anti-inflammatory and antioxidant dietary patterns yielded the greatest synergistic protective effect [104].

### Lipid metabolism and atherosclerosis

Despite the widespread use of statins, individuals with CKM syndrome continue to face a significant residual risk of atherosclerotic cardiovascular disease (ASCVD). This risk partly stems from an atherogenic lipoprotein profile not fully captured by traditional lipid metrics like LDL-C, including triglyceride-rich lipoproteins and their remnants, as well as small, dense LDL particles [87]. Emerging lipid-derived indicators provide novel perspectives for quantifying this residual risk and identifying high-risk individuals.

#### Atherogenic index of plasma (AIP) and non-HDL-C/HDL-C ratio (NHHR)

AIP, calculated as log₁₀(triglycerides/HDL-cholesterol), is a sensitive indicator of lipoprotein particle size and atherogenic potential. An elevated AIP is closely associated with adverse outcomes in CKM. First, regarding CVD risk, AIP level shows a significant positive correlation, with metabolic syndrome serving as an important mediating factor [107]. The long-term trajectory of AIP holds crucial prognostic information; analysis of the CHARLS cohort showed that individuals with poorly controlled AIP over time had a significantly higher risk of developing CVD compared to those with well-controlled levels [108]. Second, AIP demonstrates important value in predicting stroke. Evidence from CHARLS first reported a significant nonlinear positive correlation between AIP and stroke risk in the CKM population, identifying risk inflection points [109], and confirmed its status as an independent risk factor for new-onset stroke [110]. Modified AIP indices (e.g., AIP-waist-to-height ratio, AIP-WHtR) have shown superior predictive performance for stroke risk [111]. Finally, regarding mortality, NHANES-based evidence confirmed AIP as an independent predictor of all-cause and cardiovascular mortality in CKM, with this association being more pronounced in advanced stages [112]. Elevated AIP reflects a pro-atherogenic lipid profile which drives systemic inflammation, endothelial dysfunction, and oxidative stress. These pathways link dyslipidemia not only to atherosclerosis but also to progressive kidney injury [108, 109]. In the same NHANES cohort, kidney parameters (eGFR and UACR) were incorporated as critical covariates and staging criteria, underscoring the integration of kidney dysfunction within AIP-associated mortality risk [112]. Additionally, in a large prospective cohort of middle-aged and older Chinese adults with CKM stages 0–3, AIP levels were significantly correlated with eGFR, with kidney function parameters serving as core components in defining CKM stages 2 and 3 that encompass moderate-to-high-risk chronic kidney disease [107]. These findings collectively indicate that AIP is not merely a marker of dyslipidemia but also integrates kidney risk within the holistic CKM framework.

NHHR comprehensively includes cholesterol from all atherogenic lipoproteins. Cross-sectional analysis in NHANES showed a significant positive correlation between NHHR and CKM risk, exhibiting a nonlinear relationship [113]. Subgroup analysis indicated this association was particularly significant in obese and hypertensive populations, suggesting NHHR's unique value in identifying high-risk CKM individuals with specific metabolic phenotypes. Moreover, from a kidney perspective, NHHR has been directly linked to CKD. In the same NHANES analysis, NHHR was significantly associated with the presence of CKD (defined as eGFR < 60 mL/min/1.73 m^2^ or UACR > 30 mg/g), even after extensive multivariable adjustment [113]. This association is mechanistically plausible: elevated NHHR reflects a pro-atherogenic lipid burden that contributes to kidney microvascular damage, glomerular hypertension, and progressive kidney function decline.

#### Remnant cholesterol (RC)

RC, representing the cholesterol content within triglyceride-rich lipoproteins, is an ASCVD risk factor independent of LDL-C, and its importance in CKM syndrome is increasingly recognized [86, 87]. A CHARLS-based prospective study found that higher baseline RC levels were not only independently associated with the risk of advanced CKM stages (3–4) but also predicted future cardiovascular event risk in individuals at stages 0–3. Over a median follow-up of 9 years, those in the highest RC quartile had an approximately 19% increased risk compared to the lowest quartile [86]. Furthermore, the Residual Cholesterol Inflammation Index (RCII), combining RC with inflammatory markers, was identified as an independent risk factor for frailty progression in CKM patients, especially in early stages [94]. This indicates that RC-driven lipid metabolism disorder is an important driver of disease progression even in early CKM, and its monitoring may provide a new window for intervention [86, 87].

### Aging and epigenetics

Chronological age is a major risk factor for CKM syndrome, yet individual variation is substantial. Biological aging—the progressive decline in cellular and organ function—is a core driver of CKM development and progression [117, 118]. Research indicates that multiple risk factors, such as a pro-inflammatory diet [28], periodontitis [29], and environmental metal exposure [80], can increase CKM risk by accelerating biological aging. Conversely, the protective effect of higher eGDR against mortality is partially mediated by slowing age acceleration [27]. Emerging epigenetic clocks and clinical biological age algorithms can quantify an individual's intrinsic aging rate, providing a novel dimension for CKM risk assessment beyond chronological age.

#### Epigenetic age acceleration (DNAmAA) and phenotypic age acceleration (PhenoAgeAccel)

DNAmAA, calculated as the difference between DNA methylation-estimated biological age and chronological age, reflects accelerated aging (Table 2). In CKM, age acceleration based on the GrimAge algorithm (GrimAA) shows strong associations with disease severity and prognosis. A study of NHANES participants found GrimAA significantly associated with more advanced CKM stages [88]. Over a 14-year follow-up, GrimAA powerfully predicted mortality; individuals in the highest GrimAA tertile had 1.95-fold, 3.06-fold, and 1.65-fold higher risks of all-cause, cardiovascular, and non-cardiovascular mortality, respectively [88]. Mediation analysis further revealed GrimAA mediates the effects of various protective factors (e.g., physical activity, healthy diet) on mortality, suggesting reducing aging burden is a key pathway for their benefits [88]. PhenoAgeAccel, derived from routine clinical parameters, is more clinically applicable (Table 2). Studies show PhenoAgeAccel is associated with CKM severity and mediates the link between lifestyle and CKM risk. For instance, a higher "Life's Essential 9" cardiovascular health score reduced advanced CKM risk by slowing PhenoAgeAccel, revealing delayed aging as a key mechanism [10]. Another study confirmed the negative correlation between eGDR and PhenoAgeAccel, with aging acceleration partially mediating the eGDR-mortality association [27]. Additionally, the protective effect of gut microbiota-related dietary patterns on CKM was also partially achieved by delaying the acceleration of phenotypic and biological age [114].

Collectively, these epigenetic and phenotypic aging biomarkers offer distinct but complementary practical value across diagnostic, prognostic, and mechanistic domains. Diagnostically, both GrimAA and PhenoAgeAccel can identify individuals with ‘hidden’ biological aging—those whose molecular or physiological age exceeds their chronological age—thereby detecting elevated risk even before overt clinical manifestations. For individuals classified as CKM stage 0–2, where conventional markers often remain normal despite accumulating multisystem injury, these aging biomarkers can reveal subclinical disease burden that would otherwise go unrecognized [27, 88]. Prognostically, these biomarkers provide incremental predictive value beyond traditional risk factors. In the NHANES cohort, GrimAA tertile stratification yielded hazard ratios for mortality (1.95 for all-cause, 3.06 for cardiovascular) that were comparable to or exceeded those of established clinical risk scores [88]. Moreover, because these biomarkers integrate cumulative effects of multiple pathophysiological processes, they offer a composite readout of multisystem deterioration that individual metabolic or inflammatory markers cannot capture. Mechanistically, aging biomarkers serve as integrators of the diverse upstream drivers of CKM. Mediation analyses have demonstrated that GrimAA and PhenoAgeAccel account for substantial proportions of the effects of lifestyle factors (e.g., physical activity, diet quality, cardiovascular health), insulin resistance (eGDR), and environmental exposures (e.g., periodontitis, dietary inflammatory potential) on CKM progression and mortality [10, 27–29]. These findings position biological aging not merely as a correlate but as a central mechanistic pathway through which diverse risk factors converge to drive CKM pathophysiology.

#### Aging clocks as complementary tools for CKM staging and prognosis

These emerging aging biomarkers provide valuable supplemental information to traditional CKM staging. They quantify the cumulative multisystem burden at molecular and physiological levels, identifying high-risk individuals who are "biologically old" despite young chronological age. As such, they offer a complementary layer of risk assessment that captures the biological consequences of disease progression beyond what clinical staging alone can provide. Integrating "aging clocks" into the CKM management framework promises more precise risk stratification. As research shows, aging acceleration itself is a strong predictor of advanced CKM stages and mortality, with assessment value comparable to specific metabolic or inflammatory markers [27, 88]. Furthermore, because epigenetic age acceleration is modifiable by lifestyle interventions—including improved diet quality, physical activity, and weight management—these biomarkers can serve as sensitive indicators for evaluating intervention effects [10, 28]. For future therapies targeting biological aging in CKM, these markers will become key efficacy biomarkers, enabling a shift from surrogate endpoints (e.g., blood pressure, glucose control) to direct measures of biological resilience.

### Sex-specific considerations in biomarker interpretation

Emerging evidence suggests that the performance and prognostic value of several CKM biomarkers may differ between men and women, reflecting underlying biological differences in adiposity distribution, hormonal influences, inflammatory responses, and age-related risk trajectories. For example, while TyG index predicts cardiovascular disease risk in both sexes, its association appears stronger in women, particularly among postmenopausal populations, potentially due to differential effects of estrogen on insulin sensitivity and lipid metabolism [119]. Similarly, eGDR has been shown to exhibit sex-specific associations with mortality, with lower eGDR conferring greater relative risk in women than in men across CKM stages [73]. Sex differences also extend to inflammatory biomarkers. Women with CKM syndrome tend to exhibit higher levels of systemic inflammatory markers such as high-sensitivity C-reactive protein and IL-6 compared with men at equivalent stages, potentially contributing to the observation that women experience greater excess mortality risk relative to men as CKM severity advances [23, 73]. Conversely, men more frequently present with subclinical atherosclerosis—reflected by elevated coronary artery calcium scores—as the dominant phenotype within advanced CKM stages, suggesting that sex-specific biomarker panels may be required to capture the predominant disease driver in each sex [120]. These sex-specific differences underscore several important considerations for clinical interpretation. First, biomarker thresholds derived from predominantly male cohorts may not generalize to women, necessitating sex-stratified validation studies to establish sex-specific reference ranges and cut-off values. Second, the relative weighting of biomarkers in risk prediction models may need to differ by sex; for instance, incorporating measures of insulin resistance (e.g., eGDR) may provide greater incremental value in women, while markers of subclinical atherosclerosis may be more informative in men [119, 120]. Third, biological aging biomarkers such as epigenetic age acceleration may capture sex-specific trajectories of multisystem decline, with potential implications for tailored intervention timing [74]. Collectively, these observations highlight that a one-size-fits-all approach to biomarker interpretation may miss critical sex-specific pathophysiological nuances, and future biomarker discovery and validation efforts should prioritize sex-stratified analyses to optimize clinical utility across all CKM patients.

Beyond their individual predictive value, these emerging biomarkers collectively enable refined phenotypic stratification that distinguishes patients with divergent trajectories of disease progression and clinical outcomes. For instance, among individuals with early-stage CKM (stages 0–2), those with low eGDR—reflecting severe insulin resistance—exhibit accelerated biological aging and a significantly higher risk of cardiovascular events compared with those with preserved eGDR, even when conventional metabolic parameters remain within normal ranges [27, 33]. Conversely, patients with elevated SIRI are more likely to experience rapid kidney function decline and progression to advanced CKM stages, suggesting that an inflammation-dominant phenotype carries distinct prognostic implications [25, 32]. Data-driven approaches to phenotype discovery—including metabolomic profiling and unsupervised clustering of clinical biomarkers—have further validated the utility of these biomarkers for risk stratification, revealing distinct CKM subtypes with markedly different complication profiles. These findings are discussed in detail in Section “The superior performance of machine learning models in risk prediction and patient stratification”, where we examine how machine learning methods can identify patient subtypes with heterogeneous molecular characteristics and prognostic trajectories [19, 20]. Collectively, this multidimensional biomarker framework enables not only earlier detection but also precise risk stratification by capturing underlying pathophysiological heterogeneity. By identifying patients with dominant disease drivers—whether insulin resistance, inflammation, lipid dysregulation, or accelerated aging—clinicians can prioritize targeted interventions and tailor monitoring intensity to individual risk profiles, moving beyond a one-size-fits-all approach to CKM management.

## Technology drivers: revolutionary platforms for biomarker discovery

The construction and validation of novel biomarker systems are propelled by cutting-edge technological platforms. From simplified sample collection to highly complex in vitro human physiological models and powerful computational science, these platforms collectively advance CKM research into a new era.

### Dried blood spot (DBS) technology: toward equitable and scalable CKM screening

DBS technology, a minimally invasive, stable, and easily transportable sampling method, offers a promising solution for large-scale CKM biomarker screening and monitoring, particularly in resource-limited settings [44]. Systematic evaluations indicate reliable performance for several key CKM-related biomarkers on DBS platforms. The assay for HbA1c has been thoroughly validated, supporting its expansion into clinical testing for diabetes management [44]. Beyond HbA1c, several emerging markers show potential. Laboratory analytical performance for apolipoprotein B, creatinine, cystatin C, and N-terminal pro-B-type natriuretic peptide in DBS samples is encouraging, though larger field studies are needed [44]. Furthermore, DBS technology provides a platform for multi-omics analyses (e.g., metabolomics), opening avenues for novel CKM biomarker discovery. While traditional lipid measures have shown suboptimal field performance, DBS technology overall represents a significant direction toward equitable, scalable CKM risk assessment, especially suitable for remote areas or large cohort studies [44].

### Multi-organ-on-a-chip and disease modeling: reconstituting human CKM in vitro

Multi-organ-on-a-chip (MOC) technology represents a paradigm shift in modeling the complex interorgan crosstalk that defines CKM syndrome. Unlike traditional two-dimensional cell cultures, which lack tissue architecture and dynamic intercellular signaling, or animal models, which may not fully recapitulate human physiology, MOC platforms co-culture human-derived cells or organoids from multiple organs—typically adipose, liver, kidney, and cardiac tissues—within interconnected microfluidic channels that simulate physiological flow, mechanical strain, and organ-specific microenvironments [121]. This architecture enables the dynamic observation of signal molecule exchange and functional responses under controlled pathological stimuli, such as high glucose, free fatty acids, or inflammatory cytokines. Mechanistically, MOC platforms have already provided novel insights into CKM pathophysiology that were previously inaccessible. Using an adipose–liver–kidney–heart microphysiological system, researchers have demonstrated that adipocyte-derived free fatty acids not only induce hepatic steatosis and inflammatory cytokine secretion (IL-6, TNF-α) but also directly impair glomerular endothelial barrier function and cardiomyocyte contractility in a paracrine-dependent manner [121]. Moreover, MOC platforms allow for the interrogation of extracellular vesicle (EV) biology; for instance, EV-associated microRNAs (miRNAs) initially identified in patient plasma—such as hsa-let-7d-5p, hsa-miR-24-3p, and hsa-miR-126-3p—can be functionally validated in MOC systems to establish their causal role in mediating kidney and cardiovascular events [43]. From a translational perspective, MOC platforms offer several advantages for biomarker discovery and therapeutic development. First, they enable the identification of organ-specific biomarker signatures that reflect disease activity at the tissue level, as opposed to systemic markers that may be diluted or confounded by other organs. Second, they allow for the testing of candidate therapeutics in a human-relevant context before advancing to clinical trials, potentially reducing the high failure rate of drugs targeting CKM pathways. Third, by incorporating patient-derived induced pluripotent stem cell (iPSC)-derived organoids, MOC systems can capture interindividual variability and model patient-specific responses to therapies, thereby supporting the development of personalized treatment strategies. Ongoing efforts to integrate MOC platforms with real-time biosensors and multi-omic readouts—including transcriptomic, proteomic, and metabolomic analyses—promise to further accelerate the pace of discovery and validation, positioning these technologies as essential tools in the CKM translational pipeline [121].

### Computational biology and multi-omics integration

The recent development of the PREVENT equations marks an evolution in CKM risk prediction. It incorporates core CKM metrics such as eGFR and BMI, and for the first time provides a separate heart failure risk prediction module. Studies show that the PREVENT equations offer more accurate prediction of ASCVD risk. Notably, they no longer include race as a biological variable but instead introduce a Social Deprivation Index, aligning better with the social determinants of health model for CKM [38, 40], thereby providing a framework for risk-based primary prevention of heart failure [39]. Furthermore, incorporating insulin resistance indicators like eGDR into the PREVENT equations can significantly improve their predictive performance for CVD risk in the CKM population [89], demonstrating the computational advantage of integrating novel biomarkers with traditional risk models.

The rise of multi-omics technologies provides a new perspective for systematically deciphering the molecular heterogeneity and mechanisms of CKM. Through metabolomics analysis, researchers can stratify CKM patients into distinct metabolic subtypes (e.g., glycerophospholipid-enriched, fatty acid-dominant), which are closely related to disease progression risk, laying a molecular foundation for precise subtyping [19]. This aligns with findings from data-driven clustering based on clinical phenotypes [20]. For mechanistic exploration, integrative proteomic and metabolomic analyses have revealed the critical role of branched-chain amino acid (BCAA) catabolism impairment in CKM pathogenesis. Significant accumulation of plasma BCAAs in patients correlates with disease severity and has been shown in animal models to directly induce kidney injury, whereas restoring BCAA catabolism through pharmacological intervention effectively ameliorates kidney pathology, highlighting the potential therapeutic value of this pathway [122]. Genomic studies elucidate the intrinsic link between metabolic defects and organ damage at the genetic level. Research has found that loss-of-function of the mitochondrial protease LACTB disrupts phospholipid metabolic homeostasis, leading to mitochondrial dysfunction and kidney injury, thereby establishing a direct genetic link between metabolic abnormality and target organ damage [41]. Building on this, epigenetics further quantifies the impact of biological aging on CKM progression, confirming that DNA methylation age acceleration is independently associated with advanced CKM stages and all-cause mortality, providing a new molecular clock for assessing disease burden [88]. Integrated analyses of genetic, epigenetic, and multi-omics data can more systematically reveal shared mechanisms through which environmental exposures (e.g., air pollution) exacerbate CKM [42]. These multi-omics discoveries collectively indicate that future CKM risk stratification and early warning will move beyond traditional clinical phenotypes. Instead, they will integrate multidimensional biological information such as genomic features and metabolic profiles to construct refined molecular subtyping systems, offering a new paradigm for achieving truly individualized and precise prevention and control [19, 20]. Continuous evaluation of the clinical utility of biomarkers remains key to driving their translation from discovery to application [123].

## Artificial intelligence and the future of clinical decision-making

With the establishment of a multidimensional biomarker system and the integration of cutting-edge technologies, AI is evolving from an auxiliary analytical tool into a core engine driving clinical decisions. By integrating complex, high-dimensional data, AI provides unprecedented solutions for achieving precise prediction, subtyping, dynamic monitoring, and personalized intervention in CKM management.

### The superior performance of machine learning models in risk prediction and patient stratification

Traditional statistical models often fall short when dealing with the multifactorial, non-linear interactions characteristic of CKM. In contrast, machine learning (ML) models, particularly ensemble learning algorithms like Random Forest and eXtreme Gradient Boosting (XGBoost), have demonstrated significant advantages in this domain.

*Advancing beyond traditional risk prediction:* Studies indicate that ML models show immense potential in integrating complex data and identifying key risk factors, significantly enhancing CKM risk prediction. For example, in investigating the association between environmental pollutants (e.g., PFOS) and CKM, ML models based on NHANES data achieved strong discriminatory performance (AUC = 0.90) and identified PFOS as the most important risk driver [45]. Similarly, when assessing novel nutritional-inflammatory indices (e.g., RAR, SIRI), ML models confirmed their outstanding value and clinical translation potential for CKM risk stratification [46]. ML frameworks have also been successfully used to build risk prediction models for cognitive impairment in early-stage CKM patients, demonstrating their ability to handle complex clinical outcomes [48]. These studies collectively show that ML not only predicts CKM risk with high predictive performance but can also autonomously identify core contributing predictors—such as specific environmental exposures or nutritional-inflammatory status—from vast variable sets, providing powerful data-driven tools for etiological exploration and targeted prevention.

*Data-driven discovery of patient subtypes:* Unsupervised ML can identify subtypes with distinct molecular characteristics within the CKM patient population. For instance, a clustering analysis based on plasma metabolomics successfully stratified CKM patients into three stable metabolic subtypes: Cluster 1 (glycerophospholipid-enriched, with generally low metabolic levels), Cluster 2 (fatty acid-dominant, with moderate metabolic features), and Cluster 3 (glycolipid-enriched, with high metabolic activity). High-risk CKM individuals were significantly enriched in Cluster 3, and substantial metabolic heterogeneity was observed even among patients all classified as CKM stage 3 [19]. Similarly, data-driven clustering based on clinical biomarkers identified five CKM phenotypes with different complication risks in the UK Biobank cohort, providing a basis for precision medicine interventions [20]. This molecular feature-based stratification surpasses simple organ-based classification, revealing core disease-driving mechanisms and laying the groundwork for subsequent targeted therapies. However, the clinical translation of these models still faces challenges regarding data quality, interpretability, and generalizability across diverse populations [47].

*Enhancing clinical trust through explainable AI*: Despite their predictive power, machine learning models—particularly ensemble methods like XGBoost and Random Forest—have historically been viewed as “black boxes,” limiting their acceptance among clinicians who require transparency in the biological drivers underlying risk predictions. To address this, explainable AI (XAI) techniques have emerged as essential tools for clinical translation. Methods such as SHAP (SHapley Additive exPlanations) and LIME (Local Interpretable Model-agnostic Explanations) decompose individual predictions into feature contributions, allowing clinicians to understand why a specific patient received a particular risk score. For example, SHAP analysis applied to XGBoost models in CKM populations has revealed that key predictors—such as eGDR, SIRI, and specific environmental exposures—align closely with established pathophysiology, thereby reinforcing clinical credibility [45, 46]. Moreover, global interpretability techniques can identify the most influential variables across a population, providing insights into disease drivers and potential therapeutic targets. By integrating XAI into clinical decision support systems, these models transition from opaque predictive tools to transparent, hypothesis-generating instruments that can be readily interpreted at the bedside, fostering clinician trust and facilitating adoption into routine practice [48]. However, while XAI methods enhance transparency, their scope and limitations warrant careful consideration. Techniques such as SHAP provide local or global explanations based on model internals, but these explanations do not inherently capture causal relationships—they describe associations learned from observational data rather than the effects of hypothetical interventions. A fundamental principle in medical modeling is that the research goal—whether descriptive, predictive, or causal—must dictate the analytical approach [124]. Predictive models, even when accurate, cannot directly answer interventional questions without explicit causal inference frameworks. As van Amsterdam et al. caution, outcome prediction models developed and validated without regard to causal aspects of treatment decision-making may cause harm when used to guide clinical decisions, despite demonstrating strong predictive performance in validation studies [125]. An additional practical consideration for clinical translation is the maintenance of machine learning models once deployed as clinical decision support systems (CDSS). Model performance is not static; it can degrade over time due to shifts in patient populations, changes in clinical practice, or updates to laboratory assays—a phenomenon known as concept drift or model decay. Notably, the very success of a prognostic model can accelerate its degradation: if an intervention guided by the model effectively improves outcomes, the relationship between predictors and the outcome may be disrupted, rendering the original model less accurate [116]. Therefore, successful implementation of AI-based CDSS in CKM requires prospective monitoring of performance metrics, scheduled model retraining with updated data, and governance structures to manage version control and clinical validation of updated models [116]. These considerations—ranging from the proper interpretation of XAI outputs, to the causal framing of interventional questions, to the infrastructure for long-term model stewardship—are essential to translating the promise of AI in CKM into sustained, real-world clinical benefit.

### From static metrics to dynamic monitoring: integration of digital biomarkers and wearable devices

CKM is a dynamically evolving disease state where a single static measurement cannot fully capture its trajectory. The convergence of AI with wearable devices and continuous monitoring technologies has the potential to give rise to a novel class of digital biomarkers.

*Harnessing the value of continuous physiological data:* Data continuously collected via smartwatches, patches, and similar devices—such as heart rate variability, nocturnal heart rate, physical activity levels, and sleep quality—can be analyzed by AI algorithms to establish individualized health baselines. Abnormal patterns deviating from this baseline may signal acute exacerbations or long-term risks in CKM. Existing research has confirmed a significant association between objectively measured physical activity levels (via accelerometers) and CKM stages, and that activity can modify CKM-related cancer and mortality risks [126], providing proof-of-concept for developing activity-based digital biomarkers. Furthermore, overall sleep quality has been confirmed to be significantly associated with advanced CKM stages [127], supporting the necessity of incorporating continuous behavioral data into CKM risk monitoring systems.

*Enabling dynamic risk trajectory mapping:* Integrating traditional circulating biomarkers with continuous digital biomarkers, AI can generate a dynamic, personalized CKM risk trajectory. This transforms clinical management from a reactive, "event-driven" model to a proactive, "trajectory intervention" model, allowing for early intervention when unfavorable trends in the risk curve first appear.

### Building clinical decision support systems: enabling biomarker-driven precision interventions

The true potential of AI lies in translating its insights into clinically actionable plans. Constructing CKM-specific Clinical Decision Support Systems (CDSS) is key to realizing this vision.

*Personalized treatment recommendations:* Future CDSS could input a patient's real-time data—including multidimensional biomarker profiles, digital biomarkers, and genomic information—into trained AI models. The system would not only provide diagnoses and prognoses but could also recommend prioritized interventions based on evidence of treatment responses from patients with similar subtypes. This facilitates a shift from "what medication can be used" to "which medication will provide the greatest benefit." Understanding and applying new cardiovascular risk assessment tools, such as the PREVENT equations, forms the foundation for building such decision-support systems [40].

*The prospect of closed-loop intervention systems:* A step beyond "decision support" is the formation of closed-loop management systems. For example, a system could automatically adjust digital behavioral intervention plans (e.g., personalized dietary suggestions, exercise reminders) based on continuous glucose monitoring (CGM) data, physical activity levels, and medication adherence via AI algorithms, while alerting healthcare providers to abnormalities. This "monitor-analyze-intervene-remonitor" closed-loop system has the potential to greatly enhance the efficiency and effectiveness of CKM management.

*Interoperability and clinical workflow integration:* While the vision of closed-loop AI-powered systems is compelling, translating it into routine practice faces formidable “last-mile” challenges. High-dimensional multi-omics data typically reside in disparate laboratory systems or research databases, lacking the semantic interoperability—standardized terminologies, structured data formats, and application programming interfaces—required for real-time integration into electronic health records (EHRs) such as Epic or Cerner. A pragmatic pathway lies in precomputed prognostic risk scores—such as proteomic or metabolomic risk scores, or composite indices like eGDR—that condense complex molecular profiles into a single, actionable metric. These scores can be generated via centralized analytical pipelines and transmitted as discrete laboratory results using existing interoperability standards (e.g., HL7 or FHIR), bypassing the need for real-time multi-omics processing within the EHR. To enable dynamic risk trajectory visualization without inducing alert fatigue, such scores should be displayed contextually—for instance, as time-series trend lines in the patient summary—with decision support triggered only when a validated threshold is crossed or a significant deviation from baseline occurs [128]. This “human-centered AI” approach augments rather than overwhelms clinician decision-making, and early evidence suggests that precomputed risk scores integrated via standard interoperability frameworks can improve risk stratification while maintaining workflow efficiency [129].

AI empowers all the aforementioned novel biomarkers and technology platforms, integrating them into an intelligent, precision management ecosystem for CKM. Moreover, Large Language Models have shown potential in assessing CKM knowledge, indicating prospects for AI application in patient education and information support [47]. This signifies our transition from passively managing CKM complications to actively shaping its disease trajectory.

## Challenges, perspectives, and the translational pathway

**Fig. 3:**
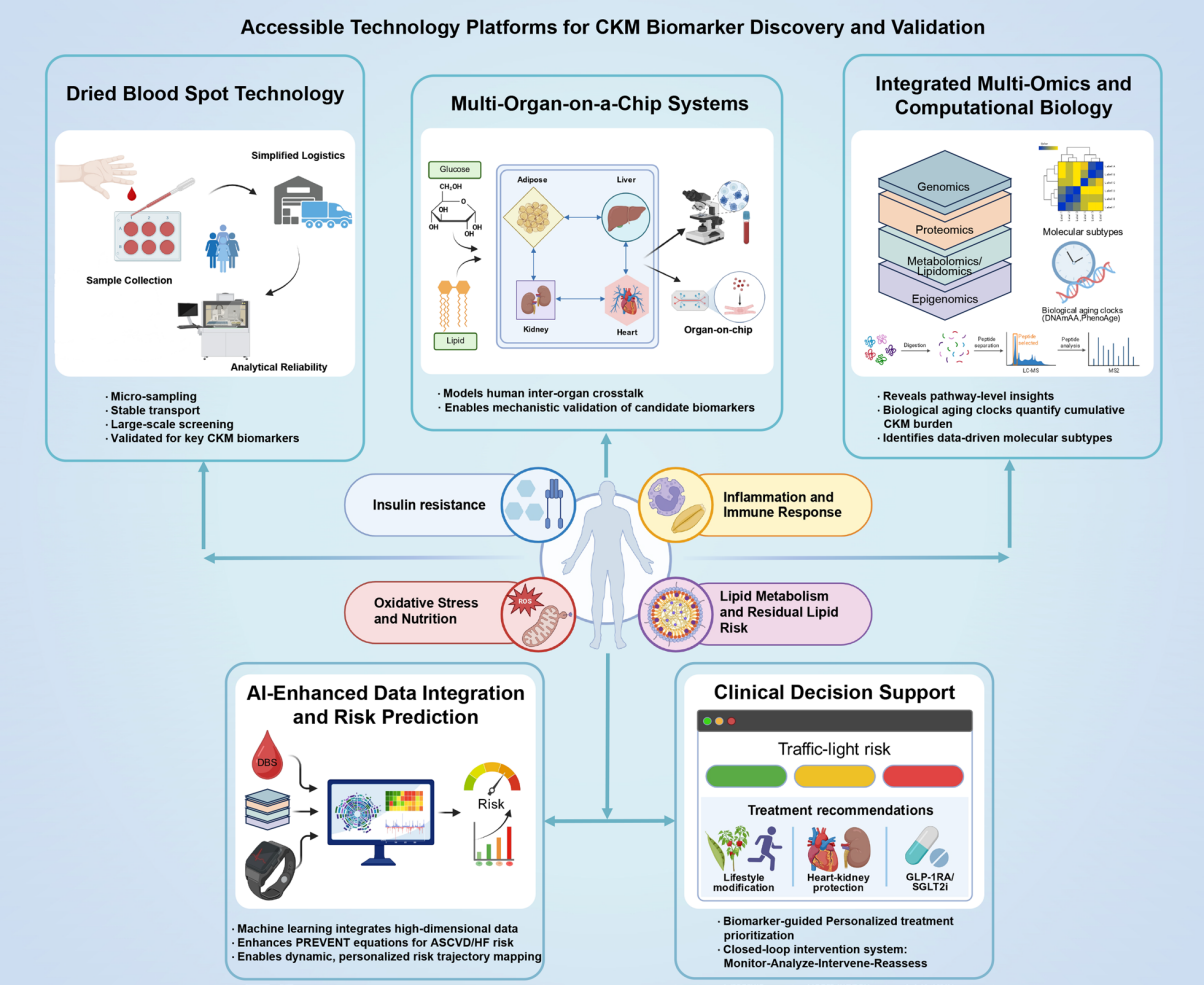
An integrative framework from biomarker discovery to precision management in Cardiovascular-Kidney-Metabolic (CKM) syndrome. This figure outlines the translational pathway, from foundational technological platforms to AI-enabled clinical decision-making. The discovery and validation of novel biomarkers are driven by three synergistic platforms: dried blood spot technology facilitates equitable, large-scale screening; multi-organ-on-a-chip systems model physiologically relevant inter-organ crosstalk; and integrated multi-omics and computational biology decode molecular heterogeneity and enable data-driven patient stratification. Collectively, these platforms underpin a multidimensional biomarker system that captures core pathological axes of CKM. Artificial intelligence serves as the central integrator, synthesizing these high-dimensional data to refine risk prediction (e.g., enhancing PREVENT equations), map dynamic risk trajectories, and power clinical decision support systems. This enables biomarker-guided therapeutic prioritization and paves the way for a proactive, closed-loop intervention system for precision management of CKM. The figure was created with BioRender.com

While the novel biomarker systems and frontier technologies hold immense promise for CKM management, their translation from research to widespread clinical practice faces multiple hurdles. A clear recognition of these obstacles and a well-defined translational pathway are crucial for realizing a paradigm shift in CKM diagnosis and treatment (Fig. 3).

### Current challenges: standardization, accessibility, and evidence hurdles

*Lack of standardization and validation:* A fundamental tension exists between biomarker discovery and the analytical standardization required for widespread clinical adoption. Unlike serum creatinine or cholesterol—which benefit from established reference materials, certified measurement procedures, and international harmonization efforts—most emerging CKM biomarkers are composite indices or multi-omics signatures that lack such infrastructure. For example, while eGDR and TyG indices have shown robust prognostic value, their derivation relies on variables measured across diverse platforms with variable accuracy, leading to substantial heterogeneity in calculated values and reference ranges across populations. Similarly, inflammatory indices such as SIRI are susceptible to variability in automated cell counting methods across different laboratories. Consequently, these biomarkers currently lack internationally unified detection or calculation standards, and their cut-off values vary by age, sex, and ethnicity, necessitating large-scale, multi-center studies to define population-specific thresholds and establish traceable reference methods. Moreover, most markers originate from observational studies, and their causal links and clinical utility still require final confirmation through prospective randomized controlled trials. Transitioning them from “research-only” to “clinically actionable” will therefore require a multi-step process: (i) consensus on standardized calculation formulas, (ii) establishment of certified reference materials, (iii) multi-center validation to define robust cut-offs, and (iv) integration into routine laboratory quality assurance programs. The recent validation framework for the PREVENT equations, which demonstrated how incorporating novel markers like eGDR can improve risk prediction while maintaining standardized input variables, offers a valuable template for this translational pathway [38, 89]. Yet, for most emerging CKM biomarkers, this translational pathway remains to be systematically established.

*Technological accessibility and health equity:* Multi-omics testing, multi-organ-on-a-chip systems, and complex AI models are costly and may exacerbate healthcare inequities. Ensuring that accessible technologies like dried blood spots reach resource-limited settings, and guaranteeing the generalizability and fairness of AI algorithms across diverse sociodemographic groups, are imperative issues that must be addressed. The significant impact of social determinants on CKM burden demands that any new system incorporates a health equity perspective. Research shows that populations with a heavier burden of social risks face a higher likelihood of CKM multimorbidity, and the disease burden is disproportionately distributed among socioeconomically disadvantaged groups [18, 70]. Consequently, if new technologies and biomarkers cannot equitably benefit all populations, especially these high-risk groups, they may inadvertently widen existing health disparities. Moving beyond passive acknowledgment of health equity, the development and deployment of AI models for CKM must proactively embed principles of fairness-aware artificial intelligence. Algorithmic bias can arise when training datasets lack diversity in race, ethnicity, sex, socioeconomic status, or geographic representation, leading to models that perform well in majority populations but systematically underperform—or even cause harm—in underrepresented groups. To mitigate this risk, several strategies should be adopted during model development. First, fairness-aware algorithms—such as those incorporating equality of opportunity constraints or adversarial debiasing—can be used to explicitly reduce performance disparities across demographic subgroups. Second, SDOH should be incorporated as primary predictive variables rather than mere covariates, as exemplified by the PREVENT equations, which include the Social Deprivation Index alongside clinical metrics [38, 40]. This approach recognizes that social context is not merely a confounder but a fundamental driver of CKM risk. Third, model performance should be systematically stratified by race, ethnicity, sex, and socioeconomic status during both development and post-deployment surveillance, with pre-specified equity thresholds to detect and correct disparities. Aligning with the 2026 American Heart Association priorities, embedding health equity into the core design of AI and multi-omics systems is essential to ensure that the precision medicine revolution reduces—rather than exacerbates—existing disparities in CKM outcomes [1, 2, 130].

*Clinical integration and health economic challenges:* Integrating a large number of novel biomarkers into existing clinical workflows presents a significant challenge. This requires training healthcare professionals and redesigning electronic health record systems. Furthermore, the cost-effectiveness of these new markers and technologies needs rigorous health economic evaluation to demonstrate their effectiveness in reducing long-term complications and thereby saving overall healthcare expenditures. Currently, empirical research in this area remains scarce. Future studies must assess the incremental health benefits and economic value of integrating novel biomarkers (e.g., eGDR) into existing risk prediction models like the PREVENT equations [89].

### Future directions: from precision subtyping to dynamic intervention systems

*Defining CKM subtypes and precision therapeutics:* Data-driven cluster analyses have preliminarily revealed CKM phenotypes with varying complication risks, laying the groundwork for precision treatment [20]. Future research should strive to use integrated multi-omics data and ML methods to define and validate reproducible CKM subtypes in large global cohorts. The goal is to achieve "biomarker-guided precision therapy," matching specific subtypes with the most effective therapeutic strategies. A tangible step toward biomarker-guided precision therapy lies in leveraging the contemporary “Four-Pillar” pharmacotherapy framework—comprising renin–angiotensin–aldosterone system inhibitors (RAASi), sodium-glucose cotransporter 2 inhibitors (SGLT2i), non-steroidal mineralocorticoid receptor antagonists (ns-MRAs), and glucagon-like peptide-1 receptor agonists (GLP-1RAs)—which collectively target the interconnected metabolic, inflammatory, and fibrotic pathways central to CKM pathophysiology [2, 131]. Importantly, emerging evidence suggests that the relative efficacy of these agents varies by underlying CKM phenotype. For instance, SGLT2i demonstrate particularly robust cardiorenal protection in patients with heart failure and albuminuric chronic kidney disease, while GLP-1RAs exert pronounced benefits in obesity-dominant phenotypes through weight loss and glycemic control [84, 132]. Similarly, ns-MRAs such as finerenone have shown additive benefits in patients with diabetic kidney disease and high residual inflammatory risk [133]. Integrating multidimensional biomarker profiles—such as eGDR for insulin resistance, SIRI for inflammation, and urinary albumin-to-creatinine ratio for kidney injury—into clinical decision-making could enable phenotype-driven selection and sequencing of these four foundational therapies. By aligning therapeutic prioritization with molecular and clinical phenotypes, this approach moves beyond a “one-size-fits-all” paradigm toward truly individualized CKM management [118].

*Embracing dynamic monitoring and digital therapeutics:* The research focus should shift from "static snapshots" to "dynamic trajectories." This involves exploring the feasibility of continuously monitored biomarkers and developing AI-based digital therapeutic platforms capable of automatically adjusting intervention strategies based on real-time data (from wearables and periodic tests), ultimately forming personalized, closed-loop management ecosystems. Current evidence already indicates a significant association between objectively measured physical activity levels and CKM risk and prognosis [126], providing a basis for constructing activity-based digital interventions.

*Targeting biological aging and emerging pathways:* Given the central role of aging biomarkers in CKM, interventions targeting the aging process itself (e.g., cellular senescence, epigenetic alterations) represent a highly promising new frontier. Aging biomarkers (e.g., DNAmAA) will become key endpoints for evaluating the efficacy of these novel therapies [88]. Concurrently, new drug development targeting specific pathological pathways in CKM is emerging—for example, targeting ketohexokinase to inhibit harmful fructose metabolism [134], supplementing inorganic nitrate to address the "nitric oxide crisis" [85], and modulating hydrogen sulfide (H₂S) signaling in early life for disease reprogramming [135]. These therapies may transcend traditional symptom management to address the root causes of disease.

### Conclusion: toward a new era of multidimensional, dynamic, and AI-empowered CKM management

The complexity of CKM syndrome demands that we move beyond traditional diagnostic and therapeutic models. This review systematically outlines an emerging novel biomarker system that provides deeper and earlier insights into the disease across multiple dimensions: metabolism, inflammation, oxidative stress, lipid metabolism residual risk, and biological aging. Technological convergence acts as an accelerator for this system's development. Dried blood spot technology offers hope for equitable and scalable screening. Multi-organ-on-a-chip and multi-omics technologies provide powerful tools for uncovering mechanisms and discovering new targets. Artificial intelligence, serving as the central integrative engine, equips us with the capability to synthesize these vast and complex datasets, enabling precise prediction and personalized decision support. Looking ahead, successful translation depends on interdisciplinary collaboration—from biologists and data scientists to clinicians, public health experts, and policymakers. This vision aligns closely with the holistic, interdisciplinary management framework proposed by the AHA aimed at improving population CKM health. Through collective efforts to overcome challenges in standardization, equity, and evidence generation, we have the potential to fundamentally transform CKM management. We can shift from the current paradigm of passively managing complications to a new model characterized by early warning, precise subtyping, dynamic intervention, and proactive health promotion, ultimately alleviating the immense personal and societal burden imposed by this syndrome.

## Data Availability

No datasets were generated or analysed during the current study.
